# Avian Colibacillosis and Salmonellosis: A Closer Look at Epidemiology, Pathogenesis, Diagnosis, Control and Public Health Concerns

**DOI:** 10.3390/ijerph7010089

**Published:** 2010-01-12

**Authors:** S. M. Lutful Kabir

**Affiliations:** 1 Graduate School of Life and Environmental Sciences, Osaka Prefecture University, Osaka, Japan; 2 Department of Microbiology and Hygiene, Faculty of Veterinary Science, Bangladesh Agricultural University, Mymensingh-2202, Bangladesh; E-Mail: lkabir79@yahoo.com

**Keywords:** poultry, colibacillosis, salmonellosis, epidemiology, pathogenesis, public health

## Abstract

Avian colibacillosis and salmonellosis are considered to be the major bacterial diseases in the poultry industry world-wide. Colibacillosis and salmonellosis are the most common avian diseases that are communicable to humans. This article provides the vital information on the epidemiology, pathogenesis, diagnosis, control and public health concerns of avian colibacillosis and salmonellosis. A better understanding of the information addressed in this review article will assist the poultry researchers and the poultry industry in continuing to make progress in reducing and eliminating avian colibacillosis and salmonellosis from the poultry flocks, thereby reducing potential hazards to the public health posed by these bacterial diseases.

## 1. Introduction

Avian colibacillosis is an infectious disease of birds caused by *Escherichia coli*, which is considered as one of the principal causes of morbidity and mortality, associated with heavy economic losses to the poultry industry by its association with various disease conditions, either as primary pathogen or as a secondary pathogen. It causes a variety of disease manifestations in poultry including yolk sac infection, omphalitis, respiratory tract infection, swollen head syndrome, septicemia, polyserositis, coligranuloma, enteritis, cellulitis and salpingitis. Colibacillosis of poultry is characterized in its acute form by septicemia resulting in death and in its subacute form by peri-carditis, airsacculitis and peri-hepatitis [1]. On the other hand, *Salmonella* infection caused by a variety of *Salmonella* species is one of the most important bacterial diseases in poultry causing heavy economic losses through mortality and reduced production [2]. Avian salmonella infection may occur in poultry either acute or chronic form by one or more member of genus *Salmonella*, under the family Enterobacteriaceae [3]. Besides, motile Salmonellae (paratyphoid group) infection cause salmonellosis in chickens and have zoonotic significance.

Avian colibacillosis has been noticed to be a major infectious disease in birds of all ages. This disease has an important economic impact on poultry production worldwide. The majority of economic losses results from mortality and decrease in productivity of the affected birds [4]. Infectious bursal disease (IBD), mycoplasmosis, coccidiosis, Newcastle disease or infectious bronchitis, as well as nutritional deficiencies all predispose the birds to this disease [5]. However, faecal contamination of egg may result in the penetration of *E. coli* through the shell and may spread to the chickens during hatching and is often associated with high mortality rates, or it may give rise to yolk sac infection. On the other hand, with the great expansion of poultry rearing and farming, avian salmonellosis is the most devastating disease worldwide. The epidemiology of fowl typhoid and pullorum disease in poultry, particularly with regard to transmission from one generation to the next is known to be closely associated with infected eggs [6]. The birds that survive from clinical disease when infected at a young stage may show few signs of infection but can become carriers [7].

At slaughter, resistant strains from the gut readily soil poultry carcasses and as a result poultry meats are often contaminated with multiresistant *E. coli* [ 8–14]; likewise eggs become contaminated during laying [15]. Hence, resistant faecal *E. coli* from poultry can infect humans both directly and via food. These resistant bacteria may colonize the human intestinal tract and may also contribute resistance genes to human endogenous flora [16]. Similarly, the emergence of multidrug resistance among *Salmonella* spp. is an increasing concern. *Salmonella* serovar Hadar has been reported as one of the most resistant *Salmonella* serotypes [17–19].

Microbial food safety is an increasing public health concern worldwide. Epidemiological reports suggest that poultry meat is still the primary cause of human food poisoning [20]. Poultry meat is more popular in the consumer market because of advantages such as easy digestibility and acceptance by the majority of people [21]. However, the presence of pathogenic and spoilage microorganisms in poultry meat and its by-products remains a significant concern for suppliers, consumers and public health officials worldwide. *E. coli* and *Salmonella* has been consistently associated with foodborne illnesses in most countries of the world.

There are many poultry diseases transmissible to human, among them avian colibacillosis and avian salmonellosis are the prime concerns. But the detailed information about avian colibacillosis and avian salmonellosis in connection to the public health concerns are not available yet in one place. So, I intend to write this review article focusing on the various aspects of avian colibacillosis and avain salmonellosis in connection to the public health concerns.

## 2. Epidemiology of Avian Colibacillosis and Avian Salmonellosis

### 2.1. Epidemiology of Avian Colibacillosis

*E. coli* is a gram-negative, non-acid-fast, uniform staining, non-spore-forming bacillus that grows both aerobically and anaerobically and may be variable in size and shape. Many strains are motile and have peritrichous flagella. *E. coli* is considered as a member of the normal microflora of the poultry intestine, but certain strains, such as those designated as avian pathogenic *E. coli* (APEC), spread into various internal organs and cause colibacillosis characterized by systemic fatal disease [22,23]. *E. coli* isolates pathogenic for poultry commonly belong to certain serogroups, particularly the serogroups O78, O1, and O2, and to some extent O15 and O55 [24,25]. In domestic poultry, avian colibacillosis is frequently associated with *E. coli* strains of serotypes O78:K80, O1:K1 and O2:K1 (2- Filali E). The avian colibacillosis was found widely prevalent in all age group of chickens (9.52 to 36.73%) with specially high prevalence rate in adult layer birds (36.73%) [26].

The most important reservoir of *E. coli* is the intestinal tract of animals, including poultry. In chickens, there are about 10^9^ colony forming units (CFU) of bacteria per gram of feces and of these, 10^6^ CFU are *E. coli. E. coli* has also been commonly isolated from the upper respiratory tract. In addition, it is present on the bird’s skin and feathers. These strains always belong to both pathogenic and non-pathogenic types [27]. In the caecal flora of healthy chickens, 10 to 15% of the *E. coli* strains may belong to an O-serotype that can also be isolated from colibacillosis lesions [28]. As soon as the first hours after hatching, the birds start building up their *E. coli* flora. The bacteria drastically increase their numbers in the gut. In a single bird a large number of different *E. coli* types is present, obtained via horizontal contamination from the environment, more specifically from other birds, faeces, water and feed [29]. Moreover, rodents may be carriers of APEC and hence a source of contamination for the birds [22].

The risk for colibacillosis increases with increasing infection pressure in the environment. A good housing hygiene and avoiding overcrowding are very important. Other principal risk factors are the duration of exposure, virulence of the strain, breed, and immune status of the bird [30–34]. Every damage to the respiratory system favours infection with APEC. Several pathogens, like NDV, IBV and MG, both wildtype and vaccine strains, may play a part in this process. An unfavourable housing climate, like an excess of ammonia or dust, renders the respiratory system more susceptible to APEC infections through deciliation of the upper respiratory tract [22].

Pulsed field gel electrophoresis (PFGE) is considered to be the most reliable molecular finger-printing technique to differentiate organisms but restriction fragment length polymorphism (RFLP) is the one that is used most frequently. However, both techniques require large quantities of DNA, are time consuming, and require expensive equipment [35]. Other techniques such as ERIC–PCR and REP–PCR [36,37] and random amplification of polymorphic DNA (RAPD)–PCR [38] have been proposed as alternatives and used to characterize *Escherichia coli* isolates of avian origin [39,40]. Other molecular techniques such as ribotyping and isoenzyme profile have also been used to evaluate the clonality of avian *E. coli* [41]. Some clones are specific to APEC and a small-scale comparison of commensal and pathogenic isolates revealed that 83% of pathogenic strains belong to only five clones, whereas each of the 10 non-pathogenic strains belong to different clones [42]. On the other hand, clonal relationships were found for O2:K1 isolates from humans and chickens [43] and for O78 isolates from humans, cattle, sheep, pigs and chickens [44], indicating that these species too might act as a source of infection for chickens.

Even though certain O-types are more frequently detected in APEC than in commensal *E. coli* [45], the isolates are very heterogenous, both in their pheno- and genotype [43,45–47]. On the other hand, the prevalence of certain serotypes is linked with the geographical localisation of a flock [48].

Since avian pathogenic *E. coli* (APEC) and human uropathogenic *E. coli* (UPEC) may encounter similar challenges when establishing infection in extraintestinal locations, they may share a similar content of virulence genes and capacity to cause disease. In this regard, Rodriguez-Siek *et al.* [49] compared 200 human uropathogenic *E. coli* (UPEC) and 524 avian pathogenic *E. coli* (APEC) isolates for their content of virulence genes (Table 1), including many implicated in extraintestinal pathogenic *E. coli* (ExPEC) virulence as well as those associated with APEC plasmids for assessing the potential of APEC to cause human extraintestinal diseases and a well-documented ability of avian *E. coli* to spread to human beings, the potential for APEC to act as human UPEC or as a reservoir of virulence genes for UPEC should be considered.

Avian pathogenic *E. coli* strains are often resistant to antimicrobials approved for poultry including cephradine [66], tetracyclines [66–70], chloramphenicol [66], sulfonamides [67,69–71], amino-glycosides [68–70,72,73] and β-lactam antibiotics [66,67,69,71]. Resistance to fluoroquinolones was reported within several years of the approval of this class of drugs for use in poultry [45,71,74,75]. There is reason for concern that genes conferring resistance to extended-spectrum beta-lactams will emerge in avian pathogenic *E. coli* strains [76] and reduce the efficacy of ceftiofur, which is currently used on a limited basis in poultry breeding flocks and hatcheries. In one study, conducted at the University of Georgia, 97 of 100 avian pathogenic *E. coli* isolates were resistant to streptomycin and sulfonamide and 87% of these multiple antimicrobial resistant strains contained a class 1 integron, intI1, which carried multiple antibiotic resistance genes [70]. Multiple antimicrobial resistance traits of avian pathogenic *E. coli* have also been associated with transmissible R-plasmids [77].

### 2.2. Epidemiology of Avian Salmonellosis

Avian *Salmonella* infections are important as both a cause of clinical disease in poultry and as a source of food-borne transmission of disease to humans. Under the family of Enterobacteriaceae, the genus *Salmonella* is a facultative intracellular pathogen causing localized or systemic infections; as well as a chronic asymptomatic carrier state [78]. The etiological agent of fowl typhoid and pullorum disease is *Salmonella enterica* subsp. *enterica* serovar Gallinarum, which is divided into two distinct biovars under the serogroup D1, Gallinarum and Pullorum, which are denoted as *S. gallinarum* and *S. pullorum,* respectively [78,79]. In addition to *S. gallinarum-pullorum*, other salmonellae such as *S. enteritidis, S. panama* and *S. dublin* also belong to the serogroup D1 [79]. The various motile and non-host adapted highly invasive serotypes such as *Salmonella enteritidis* and *Salmonella typhimurium* are commonly referred to as paratyphoid salmonellae [80]. Age wise prevalence of avian salmonellosis showed highest infection rate in adult layers (53.25%) in comparison to brooding (14.55%), growing (16.10%) and pullet (16.10%) chickens [26].

Various routes of infection have been described. Oral route of infection represents the normal route of infection [81]. Although infection in newly hatched chicks by nasal and cloacal route are also considered as the important route of transmission. Chicks may be infected early by vertical transmission either from an infected ovary, oviduct or from the infected eggs during the passage through the cloacal faeces from infected or carrier hens. The birds survive from clinical disease when infected in young stage may show few signs of infection but they become carriers [82]. In adult carriers the reproductive organs are the predilection sites that often lead to the infection of ovarian follicles and as a result transovarian transmission of the disease occurs. The bacteria are passed out through the faeces and lateral spread takes place through the fecal contaminated feeds, water and litter [78].

Although more than 2,300 serotypes of *Salmonella* have been identified, only about 10% of these have been isolated from poultry [80]. Chickens are the natural hosts for the highly host adapted biovar *S. gallinarum* and *S. pullorum*, but natural outbreaks have also been reported in turkeys, guinea fowl, quail and pheasants [83]. Fowl typhoid is a peracute, acute or chronic form of disease affecting mostly adult chickens, whereas pullorum disease affects the very young chickens, mostly 2−3 weeks of age. In the adult it tends to be chronic [78,84]. Fowl typhoid is frequently referred to as a disease of adult birds; still, there are also reports of high morbidity and mortality in young chickens [85]. *S. gallinarum* can produce lesions in chicks, which are indistinguishable from those associated with pullorum disease [78]. A certain percentage of chickens that survive from the initial infection become carriers with or without presence of clinical signs and pathological lesions [83]. Crowding, malnutrition, and other stressful conditions as well as unsanitary surroundings can exacerbate mortality and performance losses due to salmonellosis, especially in young birds [86]. The potential risk factors responsible for *Salmonella* contamination of broiler-chicken flocks are summarized in Table 2.

In more recent years, the use of DNA-related techniques such as plasmid analysis [95,96], ribotyping [97–100], and PFGE [101,102] have proved to be useful in discriminating isolates of *Salmonella* species. Lapuz *et al.* [103] investigated the prevalence of *Salmonella* in four layer farms in eastern Japan between 2004 and 2006 to determine the role of roof rats (*Rattus rattus*) in the epizootology of *Salmonella enterica* subsp. *enterica* serovar Enteritidis (*S. enteritidis*) and they suggested that roof rats were carriers of *S. enteritidis* and *S. infantis* and that persistent *S. enteritidis* and *S. infantis* infections in a rat population might play an important role in the spread and maintenance of these pathogens inside the layer premises.

Fowl typhoid and pullorum disease are distributed in many countries of the world, and have economic significance [104]. They are mainly distributed in Latin America, the Middle East, the Indian subcontinent, Africa and perhaps other parts of the world [78,105]. Salmonellosis has also been reported in many countries of South-East Asia including Bangladesh [106,107], India [108,109], Pakistan [110,111] and Nepal [112]. Fowl typhoid is common in both backyard chickens and in commercial poultry [113].

*Salmonella* and other food borne pathogens acquire antibiotic resistance by random chromosomal mutations, mutation of existing genes, and through specific mechanisms such as transduction, tranformation, and conjugation [114]. These mechanisms involve transfer of drug resistant genes by means of circular DNA plasmids such as R-factor, conjugative plasmid, or chromosomal elements [115–122]. The occurrence and proliferation of antibiotic-resistant *Salmonella* in environmental samples, poultry, and other animals and humans may be due to the use of medicated feeds [123–125], the practice of dipping hatching eggs in solutions containing antimicrobial agents [126–128], routine inoculation of day-old poults with antibiotics [126–128] and treatment of other animals [129] and humans [117] with antibiotics. *Salmonella* strains of avian origin are also often resistant to variety of antimicrobials approved for poultry including tetracycline [130–133], oxytetracycline [134], penicillin [66,130–134], aminoglycosides [130,132,133], sulfisoxazole [133] and fluoroquinolones [135]. On the other hand, Manie *et al.* [136] found several strains of multiple antibiotic-resistant *Salmonella* strains in chicken.

## 3. Pathogenesis and Disease Syndromes of Avian Colibacillosis and Avian Salmonellosis

### 3.1. Pathogenesis and Disease Syndromes of Avian Colibacillosis

The mechanisms by which avian pathogenic *E. coli* cause infection are largely unknown. The virulence factors contributing to the pathogenesis of avian colibacillosis are summarized in Table 3.

Recently, Hughes *et al.* [155] described a cross-sectional study of wild birds in northern England to determine the prevalence of *E. coli*-containing genes that encoded Shiga toxins (*stx1* and *stx2*) and intimin (*eae*), important virulence determinants of STEC associated with human disease and they stated that while wild birds were unlikely to be direct sources of STEC infections, they did represent a potential reservoir of virulence genes.

APEC are responsible for a considerable number of various diseases at different ages. Neonatal infection of chicks can occur horizontally, from the environment, or vertically, from the hen. A laying hen suffering from *E. coli*-induced oophoritis or salpingitis may infect the internal egg before shell formation. Faecal contamination of the eggshell is possible during the passage of the egg through the cloaca and after laying. The latter possibility is considered as the main route of infection for the egg [22]. Before hatching, APEC causes yolk sac infections and embryo mortality. The chick can also be infected during or shortly after hatching. In these cases, retained infected yolk, omphalitis, septicemia and mortality of the young chicks up to an age of three weeks is seen [22]. Broilers may be affected by necrotic dermatitis, also known as cellulitis, characterized by a chronic inflammation of the subcutis on abdomen and thighs [22].

Swollen head syndrome (SHS), mainly a problem in broilers, causes oedema of the cranial and periorbital skin. SHS can cause a reduction in egg production of 2 to 3%, and a mortality of 3 to 4% [156]. Data on this disease are contradictory. Picault *et al.* [157] and Hafez & Löhren [158] considered SHS as a disease caused by avian pneumovirus (APV), usually followed by an opportunistic *E. coli* infection. Nakamura *et al.* [159] however reported that APEC were probably playing a significant part in the disease, but that the role of APV was not at all clear. This had been confirmed by Georgiades *et al.* [160], who did not detect APV in any of the flocks affected by SHS during a field study, but instead detected infectious bronchitis virus (IBV), avian adenovirus, avian reovirus, and Newcastle disease virus (NDV), as well as *Mycoplasma synoviae* and *M. gallisepticum* (MG).

APEC probably do not cause intestinal diseases. Nevertheless, enterotoxigenic *E. coli* (ETEC) are occasionally isolated from poultry suffering from diarrhoea [161–163] and diarrhoea was experimentally induced after intramuscular inoculation of APEC [164]. On the other hand, enteropathogenic *E. coli* (EPEC) were isolated from clinically healthy chickens [165]. In turkeys, experimentally inoculated EPEC can only cause enteritis in combination with coronavirus [166].

Layers as well as broilers may suffer from acute or chronic salpingitis [167,168]. Salpingitis can be the result of an ascending infection from the cloaca [167,168] or an infection of the left abdominal airsac [22], although Bisgaard and Dam [167] considered the latter possibility less likely than an ascending infection. Salpingitis can lead to the loss of egg-laying capacity [163]. In the case of chronic salpingitis, the oviduct has a yellowish-gray, cheese-like content, with a concentric structure [168]. In layers, salpingitis can cause egg peritonitis if yolk material has been deposited in the peritoneal cavity, characterised by a sero-fibrinous inflammation of the surrounding tissues [22].

Airsacculitis is observed at all ages. The bird is infected by inhalation of dust contaminated with faecal material, which may contain 10^6^ CFU of *E. coli* per gram [169]. This aerogenic route of infection is considered as the main origin of systemic colibacillosis or colisepticemia [33,143,170].

Septicemia also affects chickens of all ages, and is mainly described in broilers. It is the most prevalent form of colibacillosis, characterised by polyserositis [143]. It causes depression, fever and often high mortality. Although its pathogenesis has not been elucidated, several routes of infection are possible: neonatal infections [22], infections through skin lesions [171], infection of the reproductive organs [22,167,168], of the respiratory tract [33] and even infection *per os* [172]. When *E. coli* reaches the vascular system, the internal organs and the heart are infected. The infection of the myocard causes heart failure [173]. Septicemia occasionally also leads to synovitis and osteomyelitis [22,174] and on rare occasions to panophthalmia [22]. Coligranuloma or Hjarre’s disease is characterised by granulomas in liver, caeca, duodenum and mesenterium, but not in the spleen. It is a rare form of colibacillosis, but in affected flocks it may cause up to 75% mortality [22].

Further studies are needed to determine the role of newly identified putative virulence genes and genes with unknown functions as virulence markers of APEC to strengthen the current understanding of mechanisms underlying the pathogenesis of avian colibacillosis.

### 3.2. Pathogenesis and Disease Syndrome of Avian Salmonellosis

The pathogenicity of *Salmonella* depends on the invasive properties and the ability of the bacteria to survive and multiply within the cells, particularly macrophages [175]. The main site of multiplication of these bacteria is the digestive tract, which may result in widespread contamination of the environment due to bacterial excretion through feces. Following invasion through the intestinal mucosa, cecal tonsils and Peyer’s patches, the organisms are engulfed by macrophages, and through the blood stream and/or lymphatic systems, they spread to organs rich in reticuloendothelial tissues (RES), such as liver and spleen, which are the main sites of multiplication [176]. In case of inadequate body defense mechanism, they may lead to second invasion and be localized in other organs, particularly ovary, oviduct, myocardium, pericardium, gizzard, yolk sac and/or lungs [177]. In the bird challenge, *S. typhimurium* rapidly caused inflammation of the intestinal mucosa, but *S. pullorum* preferentially targeted the bursa of Fabricius prior to eliciting intestinal inflammation [178]. Pullorum disease manifests itself predominantly as an enteric disease of chickens, while fowl typhoid shows signs of septicemic disease [78]. Both biovars can cause septicemic infections, which may be acute or chronic, but unlike *S. pullorum*, *S. gallinarum* is capable of producing peracute infection and hemolytic anemia in both young and adults [84]. *S. gallinarum* is extremely pathogenic to young broiler chicks [179].

Fowl typhoid is indistinguishable from pullorum disease unless the etiological agent is isolated and identified [113]. Clinical signs in chicks and poults include anorexia, diarrhea, dehydration, weakness and high mortality [83]. In mature fowls, fowl typhoid and pullorum disease are manifested by anorexia, drop in egg production, increased mortality, reduced fertility and hatchability [83]. *S. pullorum* infected adult birds may or may not exhibit any clinical signs, or they cannot be detected by their physical appearance [78]. Furthermore, the exact mechanisms of getting these poultry diseases are still remained to be obscured.

## 4. Diagnosis of Avian Colibacillosis and Avian Salmonellosis

Colibacillos is suspected based on the clinical features and the typical macroscopic lesions. The diagnosis is obtained by *E. coli* isolation from cardiac blood and affected tissues, like liver, spleen, pericard or bone marrow. Experimentally it was shown that in acute cases, isolation is possible from six hours to three days after infection; in subacute cases, isolation is only possible until seven days after infection [180]. Contamination from the intestines is rarely a problem, if fresh material is used and standard bacteriological procedures are applied [181]. Selective media like McConkey, eosin-methylene blue or drigalki agar are used for isolation. Further identification of the isolated colonies is based on biochemical reactions (indol production, fermentation of glucose with gas production, presence of ß-galactosidase, absence of hydrogen sulphite production and urease, and the inability to use citrate as a carbon source) [29]. O-serotyping is a frequently used typing method. An ELISA, based on sonicated *E. coli*, has been developed for detection of antibodies against two important pathogenic serotypes of *E. coli*: O78:K80 and O2:K1 [182]. Another ELISA was based on fimbrial antigen [183]. Both have limited value because they can only detect homologous APEC types. All currently known virulence-associated factors, detected in strains isolated from colibacillosis lesions, can also be detected in faecal isolates from clinically healthy chickens. For this reason, none of these traits can be used for APEC identification.

Diagnosis of avian salmonellosis should be confirmed by isolation, identification, and serotyping of *Salmonella* strains. Infections in mature birds can be identified by serologic tests, followed by necropsy evaluation complemented by microbiologic culture and typing for confirmation. A serological ELISA test for the diagnosis of avian salmonellosis either with *S. typhimurium* or *S. enteritidis* has been established [184]. Szmolka *et al.* [185] established a diagnostic and a real-time PCR system for rapid and reliable genus- and serovar- (*S. enteritidis* and *S. typhimurium*) specific detection of *Salmonella* for monitoring purposes in the poultry food chain.

## 5. Preventive Measures for Controlling Avian Colibacillosis and Avian Salmonellosis

### 5.1. Avian Colibacillosis

A first step is the prevention of egg contamination by fumigating them within two hours after lay, and by removing cracked eggs or eggs soiled with faecal material. It is recommended to vent the incubators and hatchers to the outside and to have as few breeder flocks as possible per breeding unit [22]. In chicks, contamination with APEC from the environment must be controlled by reduction and control of intestinal infection. This can be achieved using competitive exclusion (CE) [186–190], *i.e.*, inoculating day-old chicks with normal bacterial flora of healthy adult chickens or a monoculture, for instance of *Bacillus subtilis*. Birds also need to be protected against pathogens that promote infections with APEC. This is possible by using *Mycoplasma-*free birds [22] and protecting the birds against mycoplasmas and viral diseases by vaccinations [170]. Disease introduction must also be avoided [170] by a suitable house infrastructure, the correct use of a transition zone (for changing clothes and shoes, and washing hands), and pest control: rodent faeces are a source of pathogenic *E. coli* [22]. The housing climate must be kept optimal for bird density, humidity, ventilation, dust and ammonia [29,170].

The great diversity among APEC strains limits the possibilities of vaccination, and vaccines are not used on a large scale. Several vaccines based on killed or attenuated strains have been tested experimentally. In general, they give sufficient protection against infection with homologous strains, but protection against heterologous strains is less efficient [29]. However, Melamed *et al.* [191] reported a certain degree of heterologous protection obtained with an inactivated vaccine. Passive immunisation of young birds via the breeder hens is efficient for two weeks [192], if the birds are challenged with homologous strains. Vaccines based on virulence factors like fimbriae, also give a good homologous protection, *i.e.*, against APEC possessing the same fimbriae [193].

### 5.2. Avian Salmonellosis

Although fowl typhoid and pullorum disease are widely distributed in most parts of the world, the diseases have been eradicated from commercial poultry in developed countries such as the United States of America, Canada and most countries of Western Europe [78]. Successful control programs can be achieved by developing good hygiene and management together with routine serological tests and slaughter policy [177]. The principal management procedures should include chicks free from infections, and the chicks should be placed in a cleaned, sanitized and *S. gallinarum* and *S. pullorum* free environment with strict biosecurity measures [194]. The feed and water should be free from *Salmonella* contamination. The dead birds need to be well disposed. Adequate precautions are needed to prevent infections from mechanical carriers like footwear, human clothing, hatchery disciplines, equipments, litters, crates, trucks and processing plants [195]. Wray *et al.* [196] described that the birds need to be tested at the age of 16 weeks due to immunologic maturity, at the point of lay due to stress and two consecutive times one month apart to provide the acceptable evidence that the flock is free from fowl typhoid [177]. Kabir *et al.* [189] and Kabir [190] demonstrated the potential role of probiotics for the controlling of *Salmonella* strains of poultry via the mechanisms of competitive exclusion. Vaccines may be used to control the disease, and antibiotics can be used for the treatment of fowl typhoid and pullorum disease.

## 6. Public Health Concerns of Avian Colibacillosis and Avian Salmonellosis

*E. coli* of the O2:K1 serotype isolated from human urinary tract infections and from septicemic chickens are phenotypically highly related. A distinction between both groups was only possible by examining their plasmid contents [43]. Cherifi *et al.* [44] obtained similar results for a group of O78 isolates and concluded that chickens might be a source of septicemic human O78 infections. However, contrasting results were obtained in a study by Caya *et al.* [197]. In this study, avian *E. coli* isolates from healthy and diseased birds (airsacculitis and cellulitis) and *E. coli* strains isolated from sick humans during the same period and in the same geographical area as the avian isolates were compared. The study results suggested that these avian isolates possessed very few of the attributes required to cause disease in humans. Reversely, human isolates can be pathogenic to day-old chicks after subcutaneous inoculation. Strains tested were of the serotypes O1, O2, O18 and O78 [198]. Although O157 verotoxigenic *E. coli* (VTEC) had been detected in broilers [158], the chicken was not considered as an important reservoir for this zoonotically often reported serotype. Experimental studies showed that chickens might be functioned as a reservoir: O157 strains easily infected the young birds, even at a low dose, and persisted in the caecum for up to three months [199]. The study by Stavric *et al.* [200] showed that layers were also susceptible to colonisation by O157:H7 and other VTEC after inoculation *per os*. The intestines were increasingly colonised if increasing inoculation doses were used. The older the bird, the more restricted the colonisation and persistence were. All birds involved in the experiment remained clinically healthy. Histologically, attachment and effacement lesions were detected in the proximal caeca. Chapman *et al.* [199] reported that at that time no bacteriologically confirmed human cases of O157 infections had been observed, caused by poultry. Nonetheless, chicken meat was sometimes positive for VTEC [201,202]. In addition, Manges *et al.* [203] conducted a case-control study between April 2003 and June 2004 and they demonstrated that antimicrobial resistant, urinary tract infection (UTI) causing *E. coli* could have a food reservoir, possibly in poultry or pork. Uncontrolled, avian *E. coli* represents a serious animal welfare concern and risk to public health as it is a zoonotic organism with avian *E. coli* species known to adapt to humans.

Salmonellosis is of public health concern because most of the strains of *Salmonella* are potentially pathogenic to humans and animals. Avian salmonellosis can pose a health risk to people if exposed. Symptoms appear similar to food poisoning, such as diarrhea and acute gastroenteritis. However, it appears that birds mainly acquire the disease from the environment and that infected birds play a relatively small role in the transmission of disease to domestic animals and humans. Public health concerns and the potential for foodborne zoonotic transmission have made *Salmonella* the subject of numerous international, national, and local surveillance programs [204].

## 7. Strategies for Reducing Public Health Hazards

The risk of colibacillosis can be reduced through simple precautions.

- By thorough cleaning of poultry houses.
- By ensuring proper ventilation of the poultry houses and chlorination of drinking water.
- By washing hands carefully before and after food preparation and after toileting.
- By avoiding eating raw or undercooked poultry.
- By wrapping fresh meats in plastic bags at the market to prevent fluids from dripping on other foods.
- By ensuring the correct internal cooking temperature especially when using a microwave.

The risk of salmonellosis can be also reduced through simple precautions.

- By washing hands carefully before and after food preparation and after toileting or changing diapers.
- By avoiding eating raw or undercooked eggs (or foods made with raw eggs) and poultry.
- By wrapping fresh meats in plastic bags at the market to prevent fluids from dripping on other foods.
- By ensuring the correct internal cooking temperature especially when using a microwave.
- By avoiding chicks and ducklings as pets for small children.

## 8. Conclusions

Avian colibacillosis and salmonellosis are considered to be the major bacterial disease problems in the poultry industry world-wide and these diseases constitute a major public health burden and represent a significant cost in many countries. The economic and public health burden of these diseases have made this topic time demanding. It is suggested from this review article that more effective application of existing control methods would greatly reduce the hazards to public health.

## Figures and Tables

**Table 1.** ExPEC/APEC genes used in virulence genotyping*.

**Gene**	**Description**	**Reference**
**pTJ100-related genes**		
cvaC**+**	Structural gene for the colicin V operon	[50]
iroN**±**	Catecholate siderophore receptor gene	[51]
iss**+**	Increased serum survival gene	[52,53]
iucC**±**	Involved in aerobactin synthesis	[54,55]
iutA**±**	Ferric aerobactin receptor gene; iron transport	[55]
sitA**±**	Putative iron transport gene	[56]
traT**+**	Outer membrane protein gene; surface exclusion; serum resistance	[57,58]
tsh**≠**	Temperature-sensitive hemagglutinin gene	[59]
**Iron-Related**		
feoB	Gene which mediates ferric iron uptake	[56]
ireA	Encodes an iron-responsive element;	[60]
	putative sideropohore receptor gene	
irp-2	Iron repressible gene associated with yersiniabactin synthesis	[61]
**Toxins**		
hlyD	Transport gene of the hemolysin operon	[62]
**Miscellaneous**		
fliC (H7)	Produces-flagellin protein associated with the H7 antigen group	[63]

* Descriptions of genes encoding components of certain adhesins (*i.e.*, genes encoding parts of the P pilus, papA; papC; papEF; papG, including papG alleles I, II, and III; the S pilus, sfa and the gene encoding the S fimbrial tip, sfaS; the Type 1 fimbrial adhesin, fimH; the F1C fimbrial tip, focG; and other genes encoding portions of miscellaneous adhesins, iha; afa; gafD; and bmaE); toxins (cnf-1 and cdtB); protectins (kpsMT K1; kpsMT II; kpsMT III; and rfc); siderophores (fyuA); and other miscellaneous structures (ibeA; ompT; and PAI(CFT073), a fragment from archetypal UPEC strain CFT073) can be found in Johnson and co-workers [64]. Also, the description of papG allele I’ can be found in Johnson and Stell [65].

+ These genes are listed as pTJ100-related, but they could also be listed as protectins.

± These genes are listed as pTJ100-related, but they could also be listed with the iron-related genes.

≠ These genes are listed as pTJ100-related, but they also could be listed in the miscellaneous group.

**Table 2.** A list of risk factors responsible for *Salmonella* contamination of broiler-chicken flocks.

**Risk factors**	**Reference**
Inadequate level of hygiene	[87,88]
Salmonella contamination of the previous flock with a persistence inside the house	[89,90] [91]
Contaminated day-old chicks and feed	[89,92–94]
The farm structure (>3 houses on the farm)	[89]
Wet and cold season	[89]
Litter-beetle infestation of the house	[91]

**Table 3.** A list of virulence factors contributing to the pathogenesis of avian colibacillosis.

**Virulence facors**	**Reference**
F (type 1) and P fimbrial adhesins	[137–140]
Curli	[141,142]
Factors contributing to adhesion, resistance to immunologic defense, survival in physiologic fluids, and cytotoxic effects	[143]
Factors conferring resistance to serum and phagocytosis	[138,140,144,145]
Aerobactin siderophores	[138,146]
*hylE*, a hemolysin gene	[147]
The *tsh* gene encoding temperature sensitive hemagglutinin	[141,148]
K1 Capsular antigen	[149]
Cytotoxins	[150–152]
Outer membrane proteins	[153]
Coligenicity	[151]
The heat-labile chick lethal toxin (CLT)	[154]
Verotoxin-2 like toxin	[152]
